# Book Reviews

**Published:** 1900-11

**Authors:** 


### BOOK REVIEWS, PAMPHLETS, EXCHANGES.

### BOOK REVIEWS.

[Contributions solicited for review.]

PULMONARY TUBERCULOSIS. ITS MODERN PROPHYLAXIS AND THE TREATMENT IN SPECIAL INSTITUTIONS AND AT HOME. Alvarenga Prize Essay of the College of Physicians of Philadelphia for the year 1898, revised and enlarged. By S. A. Knopf, M.D. (Paris and Bellevue, N. Y.), Physician to the Lung Department of the New York Throat and Nose Hospital; Former Assistant Physician to Professor Dettweiler, Falkenstein Sanatorium, Germany; Vice-President of the Pennsylvania Society for the Prevention of Tuberculosis; Fellow of the American Academy of Medicine; Laureate of the Academy of Medicine of Paris, etc. With descriptions and illustrations of the most important Sanatoria of Europe, the United States, and Canada. Octavo. Price, net, $3.00. P. Blackiston's Son & Co., 1012 Walnut Street, Philadelphia, Pa.

This book is an exhaustive treatise on the subject of tuberculosis and considers all phases of the question. The greater part of it is devoted to the different methods of treatment. It is a fine addition to the literature of a disease that now more than ever is engaging the thoughtful attention of the profession.

---

AN AMERICAN TEXT-BOOK OF PHYSIOLOGY. Edited by William H. Howell, Ph.D., M.D., Professor of Physiology in the Johns Hopkins University of Baltimore, Md. Second Edition. Revised. Vol. I. Published 1900, by W. B. Saunders & Co., Philadelphia. Price, Cloth, $3.00, net.

The popularity of this book is evidenced by the early issue of a second edition. The former edition was embraced in one volume, while the present has been divided into two separately bound parts and has added much to the convenience of handling the book. The first volume deals with the circulation, blood and lymph, secretion, digestion and nutrition, respiration, animal heat and the chemistry of the body. The book is written by a corps of collaborators, and contains the facts of the subject accurately set forth and well illustrated.

---

STUDIES IN SEX PSYCHOLOGY. By Havelock Ellis. The F. A Davis Co., Philadelphia, 1900.

The author is already well known as having done much work of high scientific merit in this little explored field of inquiry. The subject being under a social ban, requires delicacy of handling, and must be approached in the spirit of scientific investigation without pruriency. From this attitude alone are observations of this character to be viewed. The studies are concerned with modesty, sexual periodicity, auto-erotism, the psychological relation of the menstrual function. There is added a chapter by Perry-Coste on periodic sexual waves in the male. The work will prove of great interest to the student of obscure questions in human nature.

---

ON DIABETES MELLITUS AND GLYCOSURIA. By Emil Kleen, Ph.D., M.D. Octavo 313 pages. Price, Cloth, net $2.50. P. Blakiston's Son & Co., 1012 Walnut St., Philadelphia.

This book has been translated from the Swedish and is the result of the author's large experience with the disease while a practitioner at Carlsbad. It is designed to assist the general practitioner in acquiring of this frequent and rebellious disorder. The literature of the subject is not abundant and this volume will be a valued addition.

